# Molecular Mechanism of Vitamin K2 Protection against Amyloid-β-Induced Cytotoxicity

**DOI:** 10.3390/biom11030423

**Published:** 2021-03-13

**Authors:** Shu-Hsiang Huang, Sheng-Ting Fang, Yi-Cheng Chen

**Affiliations:** Department of Medicine, MacKay Medical College, New Taipei City 252, Taiwan; sshuang33@mmc.edu.tw (S.-H.H.); farmting@hotmail.com (S.-T.F.)

**Keywords:** vitamin K2, Alzheimer’s disease, Aβ cytotoxicity, PI3K/Akt/Bad, caspase-3, apoptosis

## Abstract

The pathological role of vitamin K2 in Alzheimer’s disease (AD) involves a definite link between impaired cognitive functions and decreased serum vitamin K levels. Vitamin K2 supplementation may have a protective effect on AD. However, the mechanism underlying vitamin K2 protection has not been elucidated. With the amyloid-β (Aβ) cascade hypothesis, we constructed a clone containing the C-terminal fragment of amyloid precursor protein (β-CTF/APP), transfected in astroglioma C6 cells and used this cell model (β-CTF/C6) to study the protective effect of vitamin K2 against Aβ cytotoxicity. Both cellular and biochemical assays, including cell viability and reactive oxygen species (ROS), assays assay, and Western blot and caspase activity analyses, were used to characterize and unveil the protective role and mechanism of vitamin K2 protecting against Aβ-induced cytotoxicity. Vitamin K2 treatment dose-dependently decreased the death of neural cells. The protective effect of vitamin K2 could be abolished by adding warfarin, a vitamin K2 antagonist. The addition of vitamin K2 reduced the ROS formation and inhibited the caspase-3 mediated apoptosis induced by Aβ peptides, indicating that the mechanism underlying the vitamin K2 protection is likely against Aβ-mediated apoptosis. Inhibitor assay and Western blot analyses revealed that the possible mechanism of vitamin K2 protection against Aβ-mediated apoptosis might be via regulating phosphatidylinositol 3-kinase (PI3K) associated-signaling pathway and inhibiting caspase-3-mediated apoptosis. Our study demonstrates that vitamin K2 can protect neural cells against Aβ toxicity.

## 1. Introduction

Alzheimer’s disease (AD) is the most common form of neurodegenerative disease [1,2]. AD patients usually experience a progressive loss of cognitive function, memory, and intellectual activity [2,3]. Amyloid-β (Aβ) peptide plays an important role in AD pathogenesis and is the main component of senile plaques [2,4,5]. Many studies have demonstrated that Aβ accumulation is a determining factor in AD [4,6,7,8,9]. The accumulation of Aβ involves a structural change from an α-helix to a β-strand and then deposition into toxic aggregated species, such as oligomers or fibrils. Toxic Aβ aggregates have been shown to cause neural cell death by apoptosis or necrosis [7,10].

Aβ-induced cytotoxicity is linked to oxidative stress [11,12], perturbation of intracellular calcium homeostasis [13], overproduction of tumor necrosis factor-α (TNF-α) [14], mitochondrial dysfunction [11,12,15] and apoptosis [10,16,17]. Determining the underlying mechanism of neuronal cell death induced by Aβ is an essential step in treating AD. The molecular mechanism underlying Aβ-induced cytotoxicity is complex. Several-signaling pathways associated with apoptosis, such as phosphatidylinositol 3-kinase (PI3K) [18], nuclear factor kappa-light-chain-enhancer of activated B cells (NF-κB) [19], mitogen-activated protein kinase (MAPK) [20], c-Jun N-terminal kinase (c-JNK) [21] and sphingomyelinase-ceramide [22] pathways in neurons, have been proposed. Several antioxidants can inhibit Aβ-induced cytotoxicity [23,24,25,26]. These include green tea extracts [23], sinapic acids [24], curcumin [25], and S-nitrosoglutathione [26]. As well, Aβ-induced apoptosis can be inhibited by several vitamins, such as vitamin A [27], vitamin C [28], and vitamin E [29].

Vitamin K represents a group of fat-soluble vitamins, including vitamins K1, K2 and K3 [30,31,32]. Vitamin K plays a crucial role in blood coagulation and bone proliferation [31,33,34], in which it is known as a cofactor of γ-glutamyl carboxylase and leads to the carboxylation of a family of proteins, vitamin K-dependent (VKD) protein [35]. The carboxylation of VKD proteins by carboxylase is to convert glutamate to γ-carboxylated glutamate through the epoxidation of vitamin K [35]. This reaction can be inhibited by warfarin, a vitamin K antagonist [36]. The physiological functions of active VKD protein include hemostasis, apoptosis, bone mineralization, calcium homeostasis, growth control, and signal transduction [31,34,35,36]. In the nervous system, vitamin K2 plays a crucial role in regulating many functions, such as sphingolipid synthesis [37,38], Growth arrest-specific protein 6 (Gas6)—associated-signaling [39,40], and cognition [41]. These functions are associated with γ-glutamyl carboxylase. In the human brain, menaquinone-4 (MK-4) is the most abundant form of vitamin K [31,32].

A possible role for vitamin K in the pathogenesis of AD was first hypothesized by Allison [42]. The dietary intake of vitamin K2 and the vitamin K2 concentration are decreased in the circulating blood of apolipoprotein E4 (ApoE4) carriers. Thus, the supplementation of vitamin K2 may have a beneficial effect in preventing or treating AD. Additionally, a study showed that a diet low in vitamin K might cause cognitive deficits in aged people [43]. We previously demonstrated that vitamin K3 analogs could effectively inhibit Aβ aggregation and reduce cytotoxicity [44].

Gas6, a protein product of the growth arrest-specific gene 6, is a VKD protein containing 11–12 γ-carboxyglutamic acid (Gla) residues [45]. Gas6 regulates several biological functions, such as cell growth, myelination, and mitogenesis, via the activation of the receptor tyrosine kinases of the Tyros3, Axl, and Mer (TAM) family [40,46]. In the nervous system, Gas6 has been shown to prevent cell apoptosis through activation of the PI3K associated protein kinase B (Akt) and Bcl-2-associated death promoter protein (Bad) (PI3K/Akt/Bad)-signaling pathway [47,48]. In Alzheimer’s disease patients, the protein level of Gas6 in the cerebrospinal fluid was found to be elevated [49]. Gas6 gene expression has been shown to activate the microglial phagocytosis via the TAM receptors [50]. The addition of Gas6 to primary cell cultures can rescue cortical neurons from Aβ-induced apoptosis [51,52]. These studies suggest that Gas6/TAM receptor-signaling may play a role in Alzheimer’s disease.

Although several studies have shown that vitamin K may have a beneficial effect on AD, the protective role and mechanism of vitamin K2 in AD remain to be elucidated. To unveil this role and molecular mechanism, we studied the cell survival rate and reactive oxygen species (ROS) formation and analyzed the related-signaling pathway using biochemical and cellular methods. C6 cell line is rat astroglia and has been used in many AD studies. Hence by using astroglioma C6 cells transfected with a C-terminal 99-residue fragment (β-CTF) of amyloid precursor protein (APP), we demonstrated that vitamin K2 could protect neural cells against Aβ-mediated apoptosis and the ROS induced by Aβ. The underlying mechanism of vitamin K2 might be via activating PI3K/Akt/Bad-signaling and inhibiting caspase-3-mediated apoptosis. The Gas6/Axl receptor may be one of the upregulation targets of PI3K/Akt/Bad-signaling by vitamin K2.

## 2. Materials and Methods

### 2.1. Construction of β–CTF/C6 Cell Line

The β-CTF fragment contains the C-terminal 99 residues (C99) of the amyloid precursor protein, the proteolytic product of APP by β-secretase, which can be further processed by γ-secretase to produce either Aβ42 or Aβ40 peptide [2]. Clones containing the β-CTF gene obtained from the PCR product of APP were constructed into a SparQ T2A lentivector (pCDH-CuO-MCS-IRES-GFP-EF1α-CymR-T2A-Puro), which is a cumate-inducible system (System Biosciences, Palo Alto, CA, USA). The plasmids containing the β-CTF gene were then transfected in 1 × 10^5^ human embryonic kidney 293 (HEK293) cells and incubated for 72 h. The harvested virus particle solution was then infected with 1 × 10^4^ astroglioma C6 cells and incubated for 72 h. Then, the C6 cell line containing the β-CTF gene (β-CTF/C6) was selected against puromycin, and the concentration was gradually increased from 10 to 150 μg/mL.

### 2.2. Real-Time PCR (qPCR) Analyses

To assay the C99 gene expression in β-CTF/C6 cells, qPCR was applied to examine the C99 gene expression. RNA was extracted from β-CTF/C6 cells treated with or without cumate by using the TRIzol reagent (Invitrogen). qPCR was performed using an ABI 7900 system (Applied Biosystems, Foster City, CA, USA) with the SYBR Green Supermix (Applied Biosystems) according to the manufacturer’s protocol. The expression of C99 was normalized to that of actin. The following PCR primers were used for C99:

Sense: 5′-GCTTCTTACCTGTGCACTTTCAG-3′.

Antisense: 5′-CTCTGGGACTAATCACCGTGCT-3′.

### 2.3. Cell Viability under Vitamin K2 Treatment

To examine the protective ability of vitamin K2 against Aβ-induced toxicity, β-CTF/C6 cells were treated with a designed concentration of vitamin K2, menaquinone-4 (MK-4) (Merck-Sigma, Darmstradt, Germany). For viability assay, 1 × 10^4^ β-CTF/C6 cells with 250 μg/mL cumate, based on the result shown in Figure S3A in supplemental information, were incubated with 0, 0.5, 1, 2.5, 5 and 10 μM of vitamin K2 (a stock solution of 500 μM MK4 dissolved in dimethyl sulfoxide) in a 96-well microtiter plate for 72 h at 37 °C in a humidified atmosphere containing 5% CO_2_. The incubated cells treated with or without vitamin K2 were then assayed for cell viability. Cell viability was assayed by adding MTT solution (10 μL) to each well, and wells were incubated for another 4−5 h at room temperature. The optical density was determined at 450 nm by using a microplate reader (FlexStation 3, Molecular Devices, Silicon Valley, CA, USA). 

### 2.4. Warfarin Assay

To test whether the protective effect of vitamin K2 is associated with carboxylation, warfarin, a vitamin K2 antagonist was added to β-CTF/C6 cells in the presence of menaquinone-4. We incubated 1 × 10^4^ β-CTF/C6 cells with 250 μg/mL cumate and 10 μM vitamin K2 (MK4) in the presence of 0–10 μM warfarin in a 96-well microtiter plate for 72 h at 37 °C in a humidified atmosphere containing 5% CO_2_. Cell viability was measured by MTT assay as described in the Cell Viability section. The effective dose of warfarin obtained in the assay was later applied for the Western blot analyses of pPI3K protein level.

### 2.5. Reactive Oxygen Species (ROS) Assay

The free radical level was measured by using the cellular reactive oxygen species detection assay kit (deep red fluorescence dye, Abcam). Essentially, 4 × 10^4^ of C6 cells were incubated in a 6-well plate with 20 μM Aβ42 and 0, 5, 10, 20 μM of vitamin K2 (MK4) for 60 h. The culture medium was replaced with a fresh medium and ROS dye for 15 min. The cultured cells in wells were then washed with phosphate-buffered saline (PBS) and treated with trypsin. After centrifugation at 1000 rpm for 5 min, the pellet was resuspended in PBS. In total, 30 μL resuspended cell solution was removed for cell counting and ROS measurement. The ROS level was measured using a flow cytometer (CytoFLEX S, Beckman Coulter Life Sciences, Indianapolis, IN, USA), with excitation/emission wavelengths of 650 nm and 675 nm for ROS. In total, 2 × 10^4^ cells for each sample were counted and used to measure ROS level.

### 2.6. Caspase Assay

A luminescence-based caspase-Glo 3/7 kit (Promega) was used to examine caspase-3 and -7 activities. Basically, 1 × 10^4^ β-CTF/C6 cells were incubated in a 96-well plate with 250 μg cumate and 0, 1 and 10 μM of vitamin K2 (MK4) for 0, 6, 18, and 24 h. For detecting caspase activity, 100 μL of the add-mix-measure reagent of caspase assay, which contains a tetrapeptide, a luminescent organic compound at the C-terminus, was added to each well and then reacted for 1 h before the luminescence measurement. The luminescence of β-CTF/C6 cells without cumate and menaquinone-4 treatment was the control.

### 2.7. Inhibitor Assay

Cultures of primary cortical neurons were prepared from 17-day-old embryos of Sprague-Dawley rats as described previously [53] and kindly provided by Prof. S.L. Yang (Institute of Translational Medicine, Chang Gung Memorial Hospital). Primary cells were cultured in 6 cm diameter dishes in minimum essential medium (Gibco) with 10% heat-activated fetal bovine serum. Following cell attachment, the culture medium was replaced with a neurobasal medium containing B-27 supplements (Invitrogen). Eight- to ten-day-old cultures in which >95% of the cells were neurons were used for further experiments.

We first examined the cell death rate of primary neurons with the treatment of Aβ42 peptides. 1 × 10^4^ primary neurons with 0, 5, 10, 20 and 40 μM of Aβ42 were cultured in a 96-well microtiter plate for 72 h at 37 °C in a humidified atmosphere containing 5% CO_2_. The cell viability was examined using an MTT assay with a similar protocol to that used in β-CTF/C6 cells. The optimized Aβ42 concentration (40 μM with cell viability ≤60% obtained from Figure S4) was then applied to examine the cell viability of primary neurons in the presence of vitamin K2 (MK4) at designated concentrations. The obtained results were later used for inhibitor assay.

To examine the possible signal pathway for the protective effect of vitamin K2 on AD, we added several inhibitors, including PD98059 (Extracellular signal-regulated protein kinases 1 and 2 (ERK) inhibitor; Sigma, USA), LY294002 (PI3K inhibitor; Sigma), A6730 (Akt 1/2 inhibitor; Sigma), SP600125 (JNK inhibitor; Sigma), SN50 (NF-κB inhibitor; Sigma), SB202190 (stress-activated protein kinase p38 (p38-SAPK) inhibitor; Sigma) and R428 (Axl inhibitor; AK Scientific, Inc., Union City, CA, USA) at designated concentrations, into 1 × 10^4^ cultured cells at 4 h before treatment with 40 μM Aβ42 and 10 μM vitamin K2 (MK4). Cultures were further incubated for 72 h, and then cell viability was measured by MTT assay.

### 2.8. Western Blot Assay

For western blot analysis, 60 μg of protein was fractioned on 12% SDS-PAGE and transferred to PVDF membranes. Following the transfer, samples were blocked with 5% nonfat milk in PBS containing 10% Tween-20 for 1 h and probed with primary monoclonal antibodies against Aβ1-16 (6E10, Abcam, 1:2000 dilution), Actin (8H10D10, Cell Signaling, 1:5000), PI3K (p85, Millipore, 1:1000) and phospho-PI3K (p85/phospho-Y607, Abcam, 1:500), Akt (11E7, Cell Signaling, 1:1000) and phospho-Akt (PKBα(Ser473), Millipore, 1:500), or Bad (D24A9, 1:200) and phospho-Bad (40A9, phosphor-Ser112, both Cell Signaling, 1:1000) overnight at 4 °C. After probing with primary antibodies, the PVDF membrane was washed three times with PBST and probed with goat anti-mouse secondary antibody (Sigma, 1:5000 dilution), then detected by using a chemiluminescence kit (GE, Pittsburgh, PA, USA). Following the manufacturer’s protocol, the detection solution was prepared by mixing a 1:1 ratio of reagent A (luminol chemicals) and B (peroxide solution). The sample blots were incubated with the detection solution for 5 min and then detected using a CCD camera image system (UVP, Rockland Immunochemical Inc., Limerick, PA, USA). The blot images were analyzed using the ImageJ program.

### 2.9. siRNA Silencing Gas6

A Gas6 siRNA Kit (58935, GE Healthcare Dharmacon, Inc., Lafayette, CO, USA) was used to knockdown Gas6. The on-target siRNA kit for Gas6 was provided with a mixture of four siRNAs. The on-target sequences for Gas6 siRNA were GAAUUUGACUUCCGUACUU, CCAUUCAGGAAACGGUCA, CUGGAUGUCGGCACGGAAA, and GCUAGAGAGGUGUUCGAGA. The off-target sequence for the control siRNA was UGGUUUACAUGUCGACUAA. 1 × 10^4^ β-CTF/C6 cells were transiently transfected with various concentrations of both on-target and off-target siRNAs using 5 µL of transfection reagent (DharmaFECT, GE Healthcare Dharmacon, Inc. USA) according to the manufacturer’s protocol. Cells were incubated in a serum-free medium for 24 h prior to the MTT viability assay.

### 2.10. Statistical Analysis

Statistical analysis of all data involved using Student’s *t*-test with original 6.0 software. Data are expressed as mean (SD). *p* < 0.05 was considered statistically significant.

## 3. Results

### 3.1. Construction of β-CTF/C6 Cell Lines

The β-CTF cell system has been successfully applied in several AD studies [54,55,56]. Following the same protocol, we constructed a β-CTF/C6 cell line and applied this system to study the effect of vitamin K2 on AD. We first examined the profile of Aβ peptide expression and cell viability of β-CTF/C6 cells. Figure S1 in supplemental information shows the mRNA profile in the presence of various cumate concentrations. The mRNA level of C99 in β-CTF/C6 cells was two- to three-fold higher than that in vector-only or the original C6 cells. We then treated β-CTF/C6 cells with cumate to examine Aβ protein expression. Both monomeric and aggregated Aβ levels were increased with increasing cumate concentration (Figure S2 in supplemental information). Results demonstrated that β-CTF/C6 cells could produce Aβ and Aβ aggregates on a cumate concentration-dependent mode. As the constructed β-CTF/C6 cells successfully produced Aβ, we examined the cell viability of β-CTF/C6 cells under Aβ induction. Cell survival decreased with increasing cumate dose (Figure S3A in supplemental information). The cell survival rate at cumate concentration ≥250 μg reduced to around 60% and showed no significant difference with the treatment of 300 μg cumate. Therefore, 250 μg cumate was chosen for the following experiments. The cell survival could further be rescued by adding γ-secretase inhibitors (Figure S3B in supplemental information). Taken together, the results demonstrated that β-CTF/C6 cells could undergo Aβ associated pathologies that induced cytotoxicity and caused C6 cell apoptosis. β-CTF/C6 cells could be a cell model for AD study.

### 3.2. Vitamin K2 Protects Cells against Aβ-Induced Cytotoxicity

**Figure 1:**
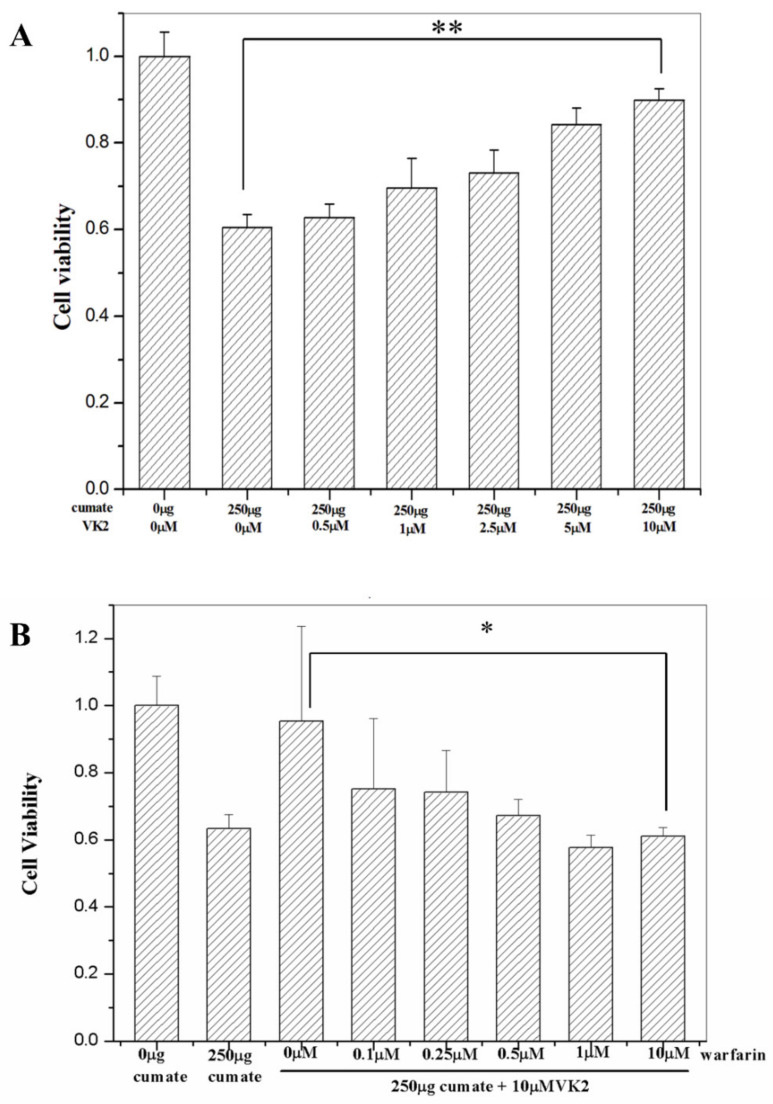
Cell viability determined by MTT assay. (**A**) Percentage survival of β-CTF/C6 cells incubated with 250 μg cumate and various vitamin K2 (VK2) concentrations for 72 h. (**B**) Warfarin reduces cell viability in the presence of vitamin K2. Percentage survival of β-CTF/C6 cells incubated with 10 μM vitamin K2 (VK2) and various warfarin concentrations for 72 h. Wells containing β-CTF/C6 cells without cumate were the control. Data are means ± SD. ** *p* < 0.01 and * *p* < 0.05.

To examine whether vitamin K2 could protect cells against Aβ-induced cytotoxicity, we first examined the cell viability of β-CTF/C6 cells in the presence of 250 μg cumate and 0, 0.5, 1, 2.5, 5, and 10 μM of vitamin K2 (Figure 1A). Cell survival was increased with increasing vitamin K2 concentration. As compared with β-CTF/C6 cell viability at 0 μM vitamin K2 and 0 μg cumate, cell viability increased from <60% at 0 μM vitamin K2 and 250 μg cumate to >90% at 10 μM vitamin K2 and 250 μg cumate. Therefore, vitamin K2 could effectively protect β-CTF/C6 cells against Aβ-induced cytotoxicity, and 10 μM vitamin K2 was used to treat cells for subsequent studies.

### 3.3. The Protective Effect of Vitamin K2 on Aβ Toxicity Is Abolished by Adding Warfarin

To further confirm the protective effect of vitamin K2 on AD, we then examined if the addition of warfarin, a well-known vitamin K2 antagonist, could abolish the vitamin K2 protection on Aβ cytotoxicity. We added 0–10 μM of warfarin in the cell culture medium with 10 μM vitamin K2 and determined the cell viability by MTT assay. The β-CTF/C6 cell viability was decreased with increasing warfarin concentration (Figure 1B). At warfarin concentration >1 μM, the cell survival rate was reduced to less than 60%, approximately the same survival rate as the control with 250 µg cumate only, indicating that warfarin could effectively diminish the protective effect of vitamin K2 on Aβ cytotoxicity. This finding confirms that vitamin K2 could effectively protect cells against Aβ-induced apoptosis.

### 3.4. Vitamin K2 Reduces the Free Radical Level

**Figure 2:**
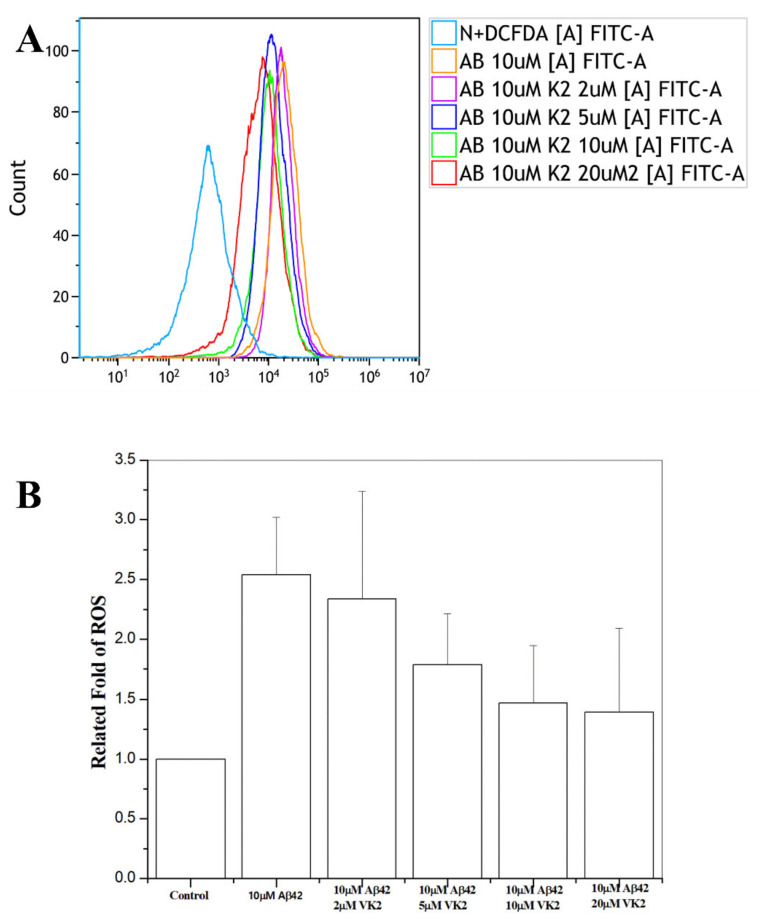
Production of reactive oxygen species (ROS) by cells. Flow cytometry of ROS production of β-CTF/C6 cells treated with (**A**) 10 μM Aβ42 and 0–20 μM of vitamin K2 (VK2) doses at 60 h. (**B**) The related statistic fold for the ROS level.

One of the mechanisms associated with Aβ cytotoxicity is the formation of free radicals [8,9]. We then examined whether the protective effect of vitamin K2 is linked with the reduction of the free radical level by using flow cytometry. By adding 2, 5, 10, and 20 μM of vitamin K2, the population of cells stained for ROS in the histogram of flow cytometer showed a decrease with an increase of vitamin K2 concentration (Figure 2A) indicating that vitamin K2 could reduce the free radicals. As compared with 10 μM Aβ42 without vitamin K2, the proportion of free radicals was 25%, 30.3%, 36.3% and 40% lower with the treatment of 2, 5, 10 and 20 μM vitamin K2 (Figure 2B), respectively. Therefore, the addition of vitamin K2 could suppress the free radical level, and vitamin K2 may protect C6 cells against Aβ toxicity by reducing free radicals.

### 3.5. Vitamin K2 Effectively Inhibit Aβ-Mediated Apoptosis

**Figure 3:**
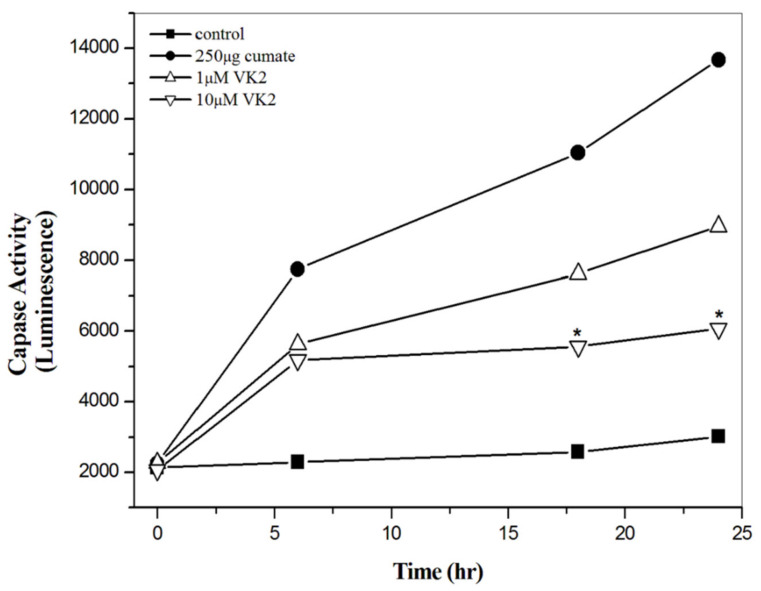
Inhibition of caspase-3 activity by vitamin K2. β-CTF/C6 cells were treated with 250 μg cumate and 0 and 10 μM of vitamin K2 (VK2). Caspase-3 activity was measured at 0, 6, 16 and 24 h. * *p* < 0.05 compared to the 250 μg cumate.

After characterizing the ROS level induced by Aβ peptides, we then examined if the protective effect of vitamin K2 could be via the inhibition of Aβ-mediated apoptosis. Since one of the apoptosis mechanisms linked with Aβ toxicity is the caspase-3 mediated apoptosis, we measured the activity of caspase-3 β-CTF/C6 cells in the presence of vitamin K2. In the absence of vitamin K2, caspase-3 activity was increased time-dependently (Figure 3). In the presence of vitamin K2, caspase-3 activity was inhibited dose-dependently. The caspase-3 activity in β-CTF/C6 cells in the presence of 1 and 10 μM vitamin K2 was about 1.2-fold and 2.5-fold lower than that in the absence of vitamin K2 at 25 h, respectively. Results suggest that vitamin K2 could significantly inhibit the caspase-mediated apoptosis induced by Aβ toxicity.

### 3.6. Vitamin K2-Mediated Protection against Aβ Toxicity Is via Activation of the PI3K/Akt Pathway

**Figure 4:**
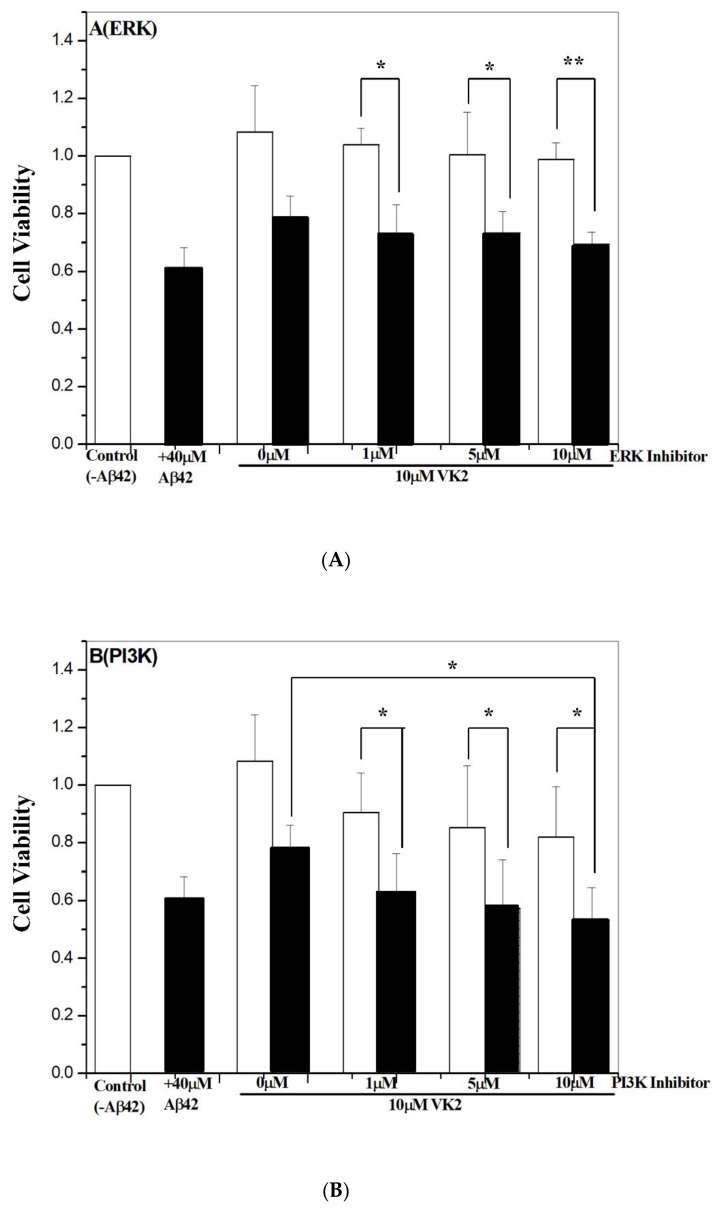

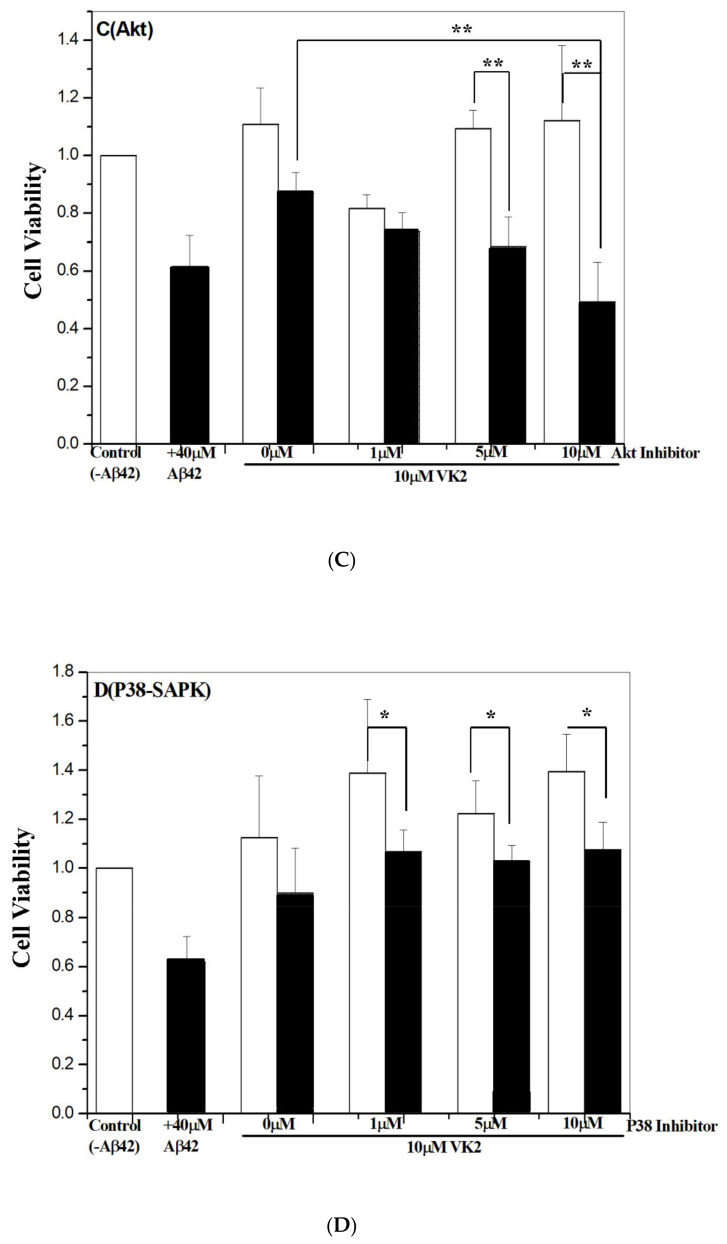

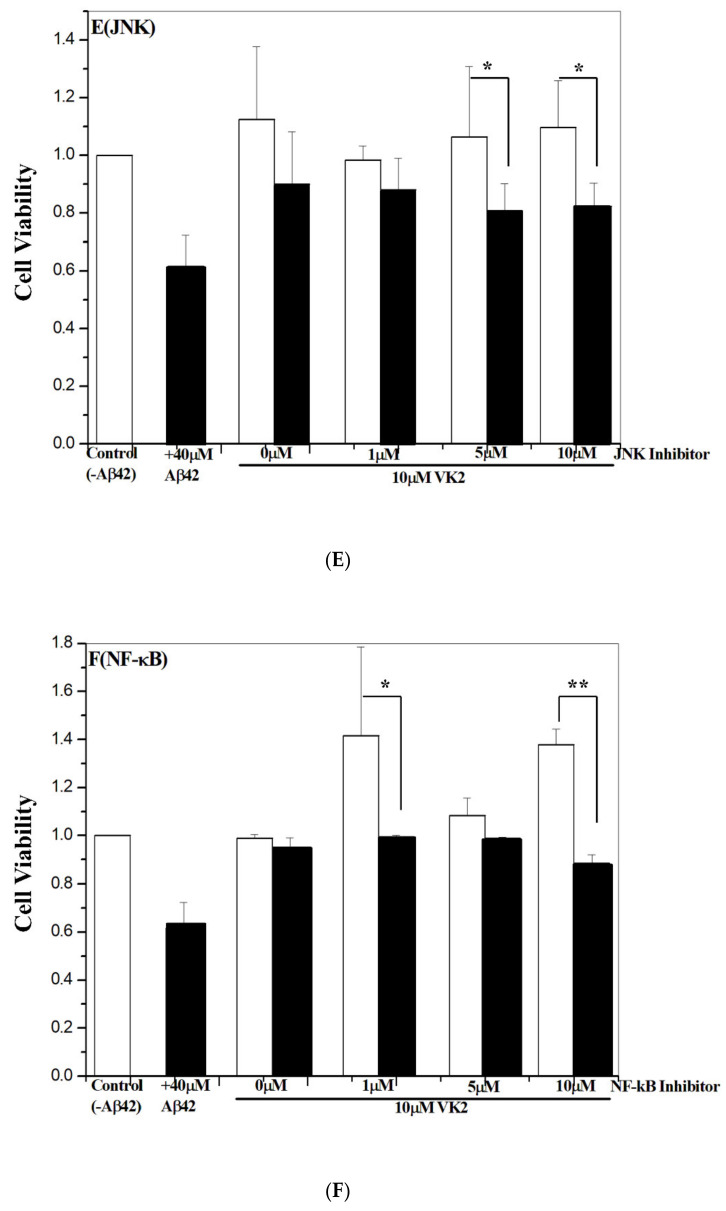
Effect of various-signaling pathway inhibitors on vitamin K2-dependent protection. Primary neurons were incubated for 72 h with 250 μg cumate, 10 μM vitamin K2 (VK2) and inhibitors for (**A**) ERK, (**B**) PI3K, (**C**) Akt, (**D**) p38-SAPK, (**E**) JNK and (**F**) Nf-κb, respectively. The cell survival rate was determined by MTT assay. Data are means ± SD. * *p* < 0.05 and ** *p*< 0.01.

Many apoptosis pathways have been linked to Aβ-induced cytotoxicity [57,58]. To examine, which apoptosis pathway is mainly associated with the vitamin K2-mediated protective effects, we used a number of different inhibitors targeted to a specific-signaling pathway. The inhibitors for the-signaling pathway study included PI3K (LY294002), Akt, ERK1/2 (PD98059), NF-κB (SN50), p38-SAPK (SB202190) and JNK (SP600125). To evaluate the inhibition without bias, the inhibition assay was performed in both primary neurons and β-CTF/C6 cells. In primary neurons with Aβ42 treatment, 60% of cell viability was obtained with a treatment of 40 μM Aβ42 (Figure S4 in supplemental information). Therefore, 40 μM Aβ42 was used for the inhibition assay in primary neurons. Related cell survival rates with the different inhibitors are shown in Figure 4A–F for primary neurons and Figure S5 in supplemental information for β-CTF/C6 cells, respectively.

As compared with controls—primary neurons treated without Aβ42 peptide—the protective effect of vitamin K2 was effectively inhibited by PI3K and Akt inhibitors, to a lesser extent by the ERK1/2 inhibitor, and not by NF-κB, p38-SPAK, and JNK inhibitors. The cell survival rate in the presence of 10 μM inhibitor was ≤60% for PI3K and Akt inhibitors, ≤70% for the ERK1/2 inhibitor, ≥80% for the JNK inhibitor, and ≥90% for NF-κB and p38-SPAK, respectively. Similar results were found with β-CTF/C6 cells (Figure S5). Thus, the molecular mechanism of vitamin K2-mediated protection may be via multiple-signaling pathways. Furthermore, the effect was more significant for PI3K and Akt inhibitors than ERK1/2, JNK, p38-SAPK, and NF-κB inhibitors, so the primary mechanism responsible for vitamin K2 protection may be via activation of PI3K/Akt-signaling.

### 3.7. Western Blot Analysis of Phosphorylated PI3K and Akt Proteins

**Figure 5:**
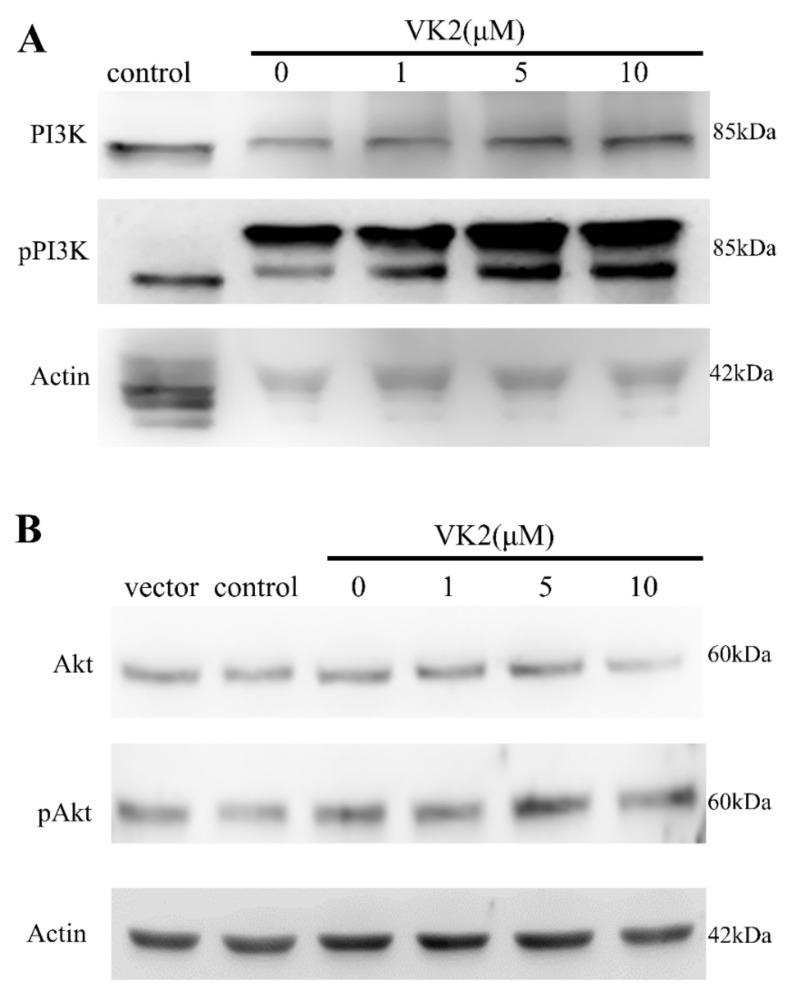

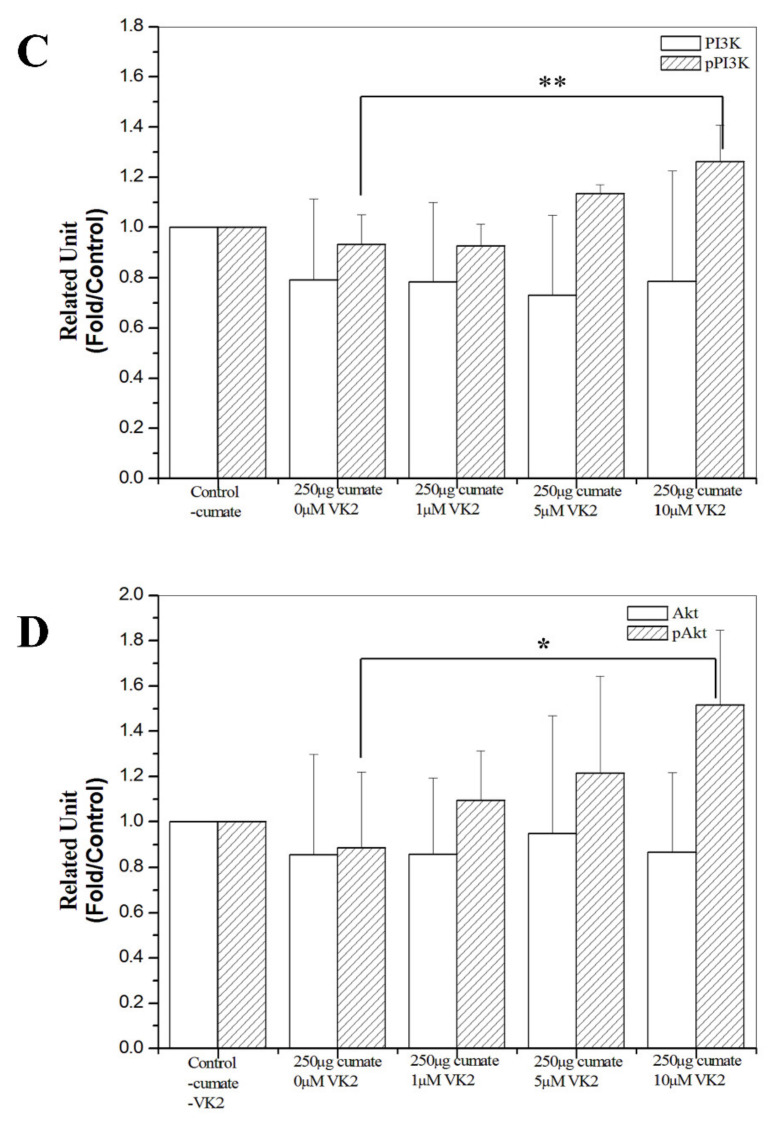
Effect of vitamin K2 on Aβ-mediated PI3K/Akt phosphorylation. β-CTF/C6 cells were treated with 250 μg cumate and 0–10 μM of vitamin K2 (VK2) for 72 h. In total, 60 μg total protein from each sample was used for Western blot analysis with monoclonal antibodies against PI3K and phosphorylated-PI3K (pPI3K) (**A**) and Akt and pAkt (**B**). Shows the relative protein level/actin for PI3K and pPI3K (**C**) and Akt and pAkt (**D**). Data are means ± SD. * *p* < 0.05 and ** *p* < 0.01.

To further confirm the role of the PI3K/Akt pathway on the protective effect of vitamin K2, we analyzed the phosphorylation of proteins in the PI3K/Akt signal pathway in β-CTF/C6 cells. Figure 5A,B shows the Western blot analyses for PI3K (pPI3K) and Akt (pAkt), respectively. The relative phosphorylated levels of PI3K and Akt are in Figure 5C,D. Phosphorylated PI3K and Akt levels were increased with increasing vitamin K2 concentration. The level of pPI3K and pAkt was 1.3- to 1.5-fold higher in cells treated with 10 μM vitamin K2 compared with the level of pPI3K and pAkt in cells without treatment of vitamin K2 (Figure 5C,D). The western analyses confirm the results obtained in the inhibitor assay, in which the protective effect of vitamin K2 against Aβ toxicity was highly associated with the activation of the PI3K/Akt-signaling pathway.

### 3.8. Warfarin Abolished Vitamin K2-Mediated Phosphorylation of PI3K

**Figure 6:**
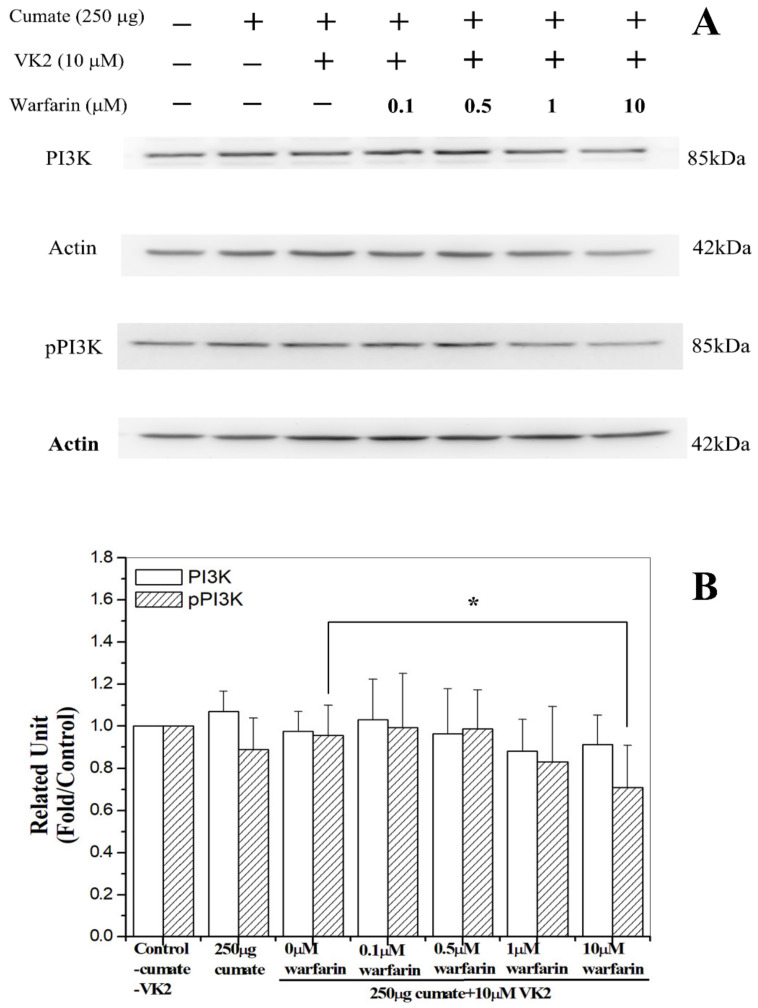
Warfarin inhibits vitamin K2-mediated PI3K phosphorylation. β-CTF/C6 cells were cultured with 250 μg cumate, 10 μM vitamin K2 (VK2), and 0–10 μM warfarin doses for 72 h. (**A**) Western blot analysis with a monoclonal antibody against PI3K and pPI3K, and (**B**) relative PI3K or pPI3K level/actin. Data are means ± SD. * *p* < 0.05.

As warfarin, the vitamin K2 antagonist could abolish the protective effect of vitamin K2 against Aβ toxicity, we then examined if the treatment of warfarin would reduce the phosphorylated level of PI3K. We treated β-CTF C6 cells with warfarin at a concentration range of 0–10 μM and measured the phosphorylated PI3K level. Basically, the phosphorylation of PI3K level was decreased with an increase in warfarin treatment (Figure 6A,B). The level of pPI3K in cells was reduced to 75% with the treatment of 10 μM warfarin. This result is consistent with the Western blot analyses. Here, our results confirm that the molecular mechanism underlying vitamin K2-mediated protective effects may be via activating PI3K/Akt-signaling.

### 3.9. Vitamin K2 Activates Antiapoptosis via Phosphorylation of Bad

**Figure 7:**
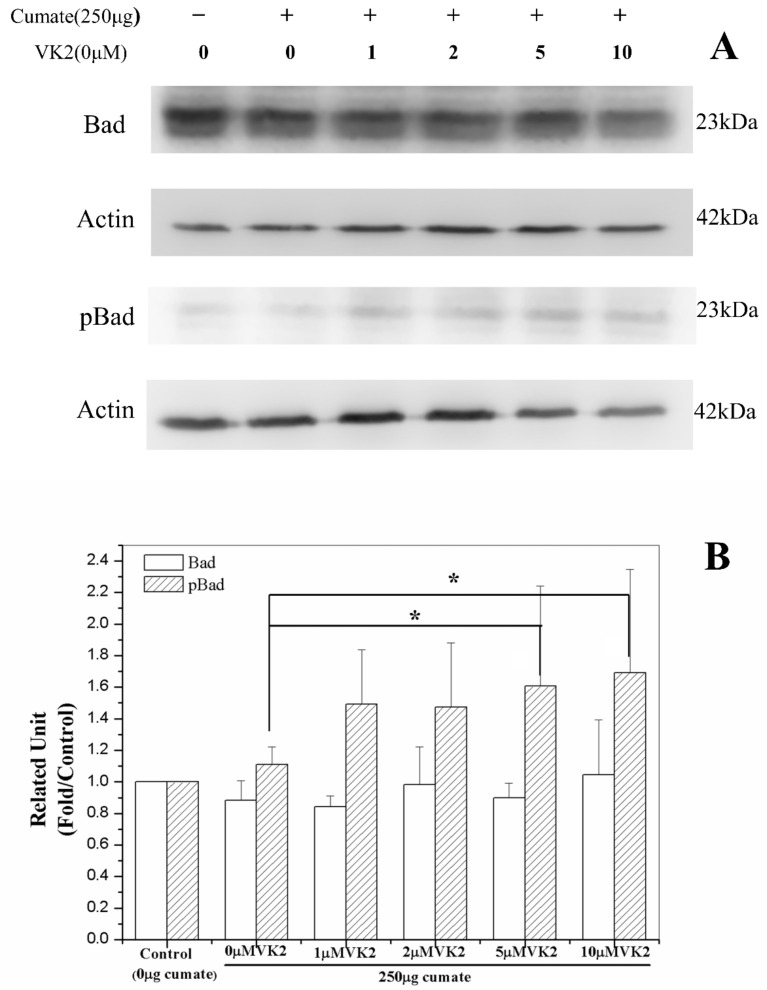
Effect of vitamin K2 on Bad phosphorylation. β-CTF/C6 cells were treated with 250 μg cumate and 0–10 μM of vitamin K2 (VK2) for 72 h. (**A**) Western blot analysis with a monoclonal antibody against Bad and p-Bad, and (**B**) relative Bad or p-Bad level/actin. Data are means ± SD. * *p* < 0.05.

The phosphorylation of Bad protein occurs secondary to the PI3K/Akt-signaling pathway and can block the caspase-3—mediated apoptosis [59,60]. Following the demonstration that vitamin K2 could protect the cells against Aβ-mediated apoptosis via the PI3K/Akt-signaling pathway, we examined whether the activation of PI3K/Akt could activate Bad-mediated antiapoptosis. Similar to the PI3K/Akt analyses, phosphorylated Bad level increased with increasing vitamin K2 concentration (Figure 7). The phospho-Bad level at vitamin K concentration ≥5 μM was 1.6–1.7-fold higher than that of the control, indicating that vitamin K2 also could activate Bad via PI3K/Akt-signaling and inhibit the caspase-3 mediated apoptosis.

### 3.10. Vitamin K2-Mediated PI3K/Akt Pathway Upregulated via Gas6/Axl Receptor

Gas6 is a VKD protein and is widely expressed in the neuron system [41]. Gas6/Axl-signaling has been shown to regulate cell survival and proliferation via the PI3K/Akt-signaling pathway [48,49]. We then examined whether Gas6 and Axl also play a role in vitamin K2 protection against Aβ cytotoxicity.

**Figure 8:**
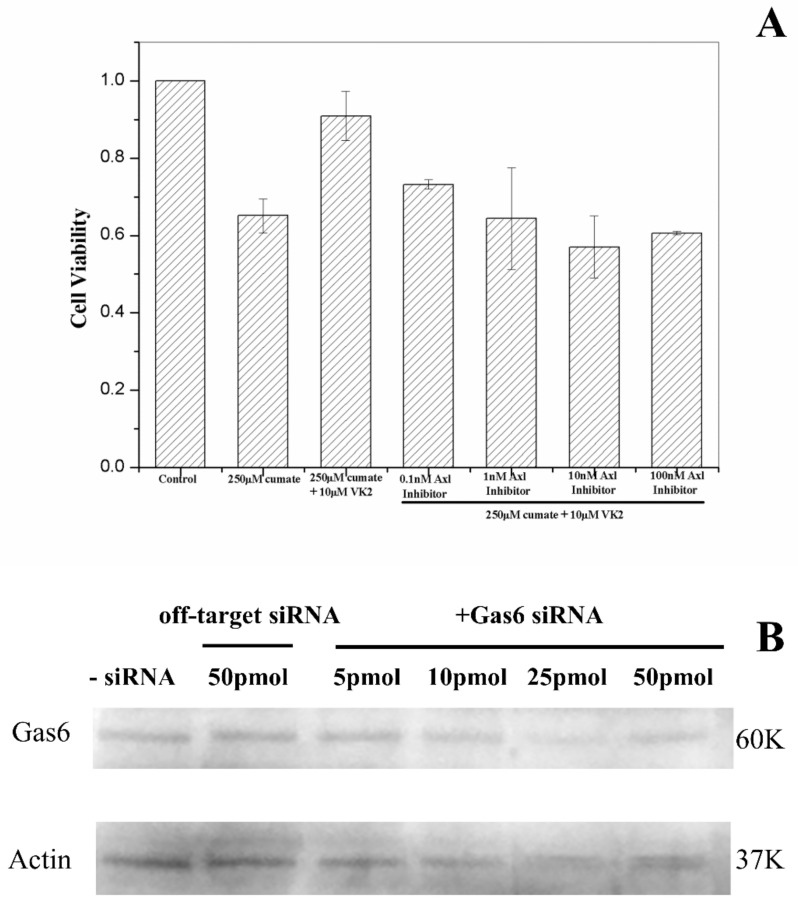
(**A**) Effect of Axl inhibitor on vitamin K2-dependent protection. β-CTF/C6 cells were incubated for 72 h with 250 μg cumate, 10 μM vitamin K2 (MK4) and various concentrations of Axl inhibitor. The cell survival rate was determined by MTT assay. (**B**) Effect of Gas6 activity on vitamin K2-dependent protection. (**A**) The knockdown of Gas6 protein treated with 5–50 pmol of Gas6 siRNA and 50 pmol of off-target siRNA.

We first investigated the role of Axl using R428, an Axl inhibitor. β-CTF/C6 cells were cotreated with vitamin K2 and 0.1–100 nM of R428. In the presence of the Axl inhibitor, the cell survival rate decreased with an increase of Axl-inhibitor concentration (Figure 8A). At the R428 concentration ≥10 nM, the cell survival rate reduced to 60%, about the same cell viability for cells that were treated with 250 μg cumate only. Results demonstrate that the protective effect of vitamin K2 in Aβ toxicity was diminished by adding an Axl inhibitor and suggest that Axl may play a role in the vitamin K2-mediated PI3K/Akt/Bad-signaling pathway.

The role of Gas6 in the vitamin K2-mediated PI3K/Akt/Bad-signaling pathway was assayed using Gas6 siRNA (Figure 8B). The knockdown level of the Gas6 protein was determined by Western blot and showed was highly dependent on the concentration of Gas6 siRNA (Figure 8B). At siRNA concentrations higher than 10 pmol, the expression level of Gas6 protein was effectively inhibited. We used 25 pmol siRNA, in which Gas6 siRNA had no significant effect on cell survival rate (control in Figure 8B), for further cell viability study. In the presence of 25 pmol Gas6 siRNA, the protective effect of vitamin K2 in Aβ toxicity was moderately inhibited (Figure 8). At the vitamin K2 concentration lower than 10 μM, the cell survival rate showed no distinct difference with or without treatment of siRNA, whereas at 10 μM vitamin K2, the cell survival rate was reduced, but not significantly by the addition of Gas6 siRNA (Figure 8). Results suggest that Gas6 may partly play a role in the regulation of the PI3K/Akt/Bad-signaling pathway induced by vitamin K2.

## 4. Discussion

Vitamin K deficiency is linked to AD in many aspects. The serum vitamin K2 level was found to be decreased in AD patients [44]. Vitamin K deficiency could cause increased cognitive deficits in aged but not young rats [42]. As well, geriatric patients who take vitamin K antagonists as anticoagulant medications show cognitive impairment [61]. Vitamin K2 supplementation had a beneficial effect in preventing or treating AD [39,43]. However, the exact effect and function of vitamin K2 in AD pathogenesis remains to be elucidated.

The β-CTF fragment has been constructed into several cell lines and used to study the inflammatory responses and synaptic dysfunction [55], the mitochondrial dysfunction [56], the endosomal enlargement and disrupted NGF-signaling [62], and the preventive effect of tricyclic pyrone compounds on C99-induced cell death [54]. To unveil the protective effect and mechanism of vitamin K2 in AD, we constructed a similar β-CTF system into astroglioma C6 cells. The β-CTF/C6 cells could overexpress Aβ peptides that induced neurotoxicity and cause neuron death [63]. When γ-secretase inhibitors were added, cell survival was increased. Taken together, this cell system can simulate the pathogenesis of AD and is suitable for AD studies.

By using the β-CTF-C99/C6 cell model, we demonstrated that vitamin K2 could effectively protect cells against Aβ toxicity. The protective effect of vitamin K2 against Aβ cytotoxicity may be attributed to the reduction of ROS and inhibition of apoptosis mediated by Aβ. The critical mechanism underlying the protective effect of vitamin K2 on Aβ-mediated apoptosis is mainly associated with the activation of PI3K/Akt-signaling, since both PI3K and Akt inhibitors were effectively reduced the cell viability with vitamin K2 treatment, and the phosphorylated level of PI3K and Akt was increased with treatment of vitamin K2 and could be inhibited by adding warfarin. This is concomitant with the result that the addition of warfarin could reduce the increase of cell viability and pPI3K protein level induced by vitamin K2. All these results demonstrate that the mechanism underlying the protective effect of vitamin K2 on Aβ-mediated apoptosis is via PI3K/Akt-signaling.

From the inhibitor assay, the inhibition of vitamin K2 protection by the other inhibitors was not as significant as that by either PI3K or Akt inhibitor, since both PI3K and Akt inhibitors were effectively reduced the cell viability with vitamin K2 treatment, and the phosphorylated level of PI3K and Akt was increased with treatment of vitamin K2 and could be inhibited by adding warfarin. This is concomitant with the result that the addition of warfarin could reduce the increase of cell viability and pPI3K protein level induced by vitamin K2. All these results demonstrate that the mechanism underlying the protective effect of vitamin K2 on Aβ-mediated apoptosis is via PI3K/Akt-signaling.

One of the cell survival signals associated with PI3K/Akt-signaling is the activation of Bad protein and caspase-mediated apoptosis [63]. In our study, we showed the phosphorylation of Bad increased with increasing vitamin K2 dose, and the caspase-3-mediated apoptosis decreased with an increase of vitamin K2 concentration, indicating that vitamin K2 inhibited the caspase-3-mediated apoptosis via activation of Bad protein. Therefore, the mechanism underlying the vitamin K2 protection against Aβ cytotoxicity is via the activation of the PI3K/Akt/Bad-signaling pathway and inhibiting the caspase-mediated apoptosis.

The PI3K/Akt-signaling pathway has been shown to play a vital role in survival signals in several neuronal cell types [19,64,65]. The function of Akt on cell survival signals has been linked to inhibiting the cytoplasmic cell death machinery and regulating the expression of genes involved in cell death and survival [64,65]. The PI3K/Akt-signaling pathway involved in the protective role against Aβ toxicity has been found associated with many natural compounds and antioxidant reagents, such as salidroside [66] and nitroso glutathione [27]. Similar to these compounds, we found that vitamin K2 could also protect neural cells against Aβ toxicity via the activation of the PI3K/Akt/Bad-signaling pathway and the inhibition of caspase-3-mediated apoptosis.

**Figure 9:**
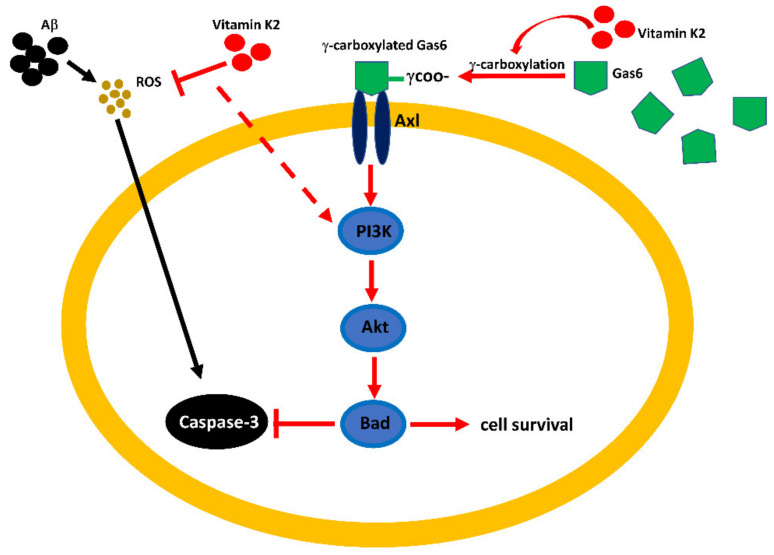
Summary of the possible molecular mechanism and signal pathway involved in vitamin K2 protection against Aβ-induced cytotoxicity.

As summarized in Figure 9, we demonstrated that both the Axl receptor and Gas6 might play a role in the mechanism underlying the protective effect of vitamin K2 on AD. Several lines of evidence have supported that Gas6 might play a role in Alzheimer’s disease. First, in the cerebrospinal fluid of patients with Alzheimer’s disease, the protein level of Gas6 was elevated [50]. Second, Gas6 is a VKD protein and is commonly expressed in the neuron system [41]. Third, Gas6/Axl receptor-signaling has been shown to regulate cell survival via the PI3K/Akt-signaling pathway [48,49]. All these factors lead us to postulate that vitamin K2 might activate Gas6 through the carboxylation of the glutamate residues. Then, the activated Gas6 might activate the PI3K/Akt/Bad-signaling pathway through the Axl receptors and protect cells against caspase-3-mediated apoptosis.

Here we described the role and mechanism of vitamin K2 in AD. Vitamin K2 had a protective effect against Aβ cytotoxicity in neural cells. The protective effect is likely to inhibit the Aβ-mediated apoptosis. The possible mechanism underlying the vitamin K2 protection is mainly via the PI3K/Akt/Bad-signaling pathway and further inhibiting caspase-3-mediated apoptosis. The activation of the PI3K/Akt/Bad pathway may partly be regulated via the Gas6/Axl receptor. Our study supports that vitamin K2 supplementation may have a beneficial effect on AD [43].

## Data Availability

Not applicable.

## Supplementary Materials

The following are available online at https://www.mdpi.com/2218-273X/11/3/423/s1, Figure S1: qPCR analysis of C99 expression vs. cumate doses, Figure S2: Western blot analyses of Aβ protein expression in βCTF/C6 cells treated with 0–250 μM of cumate, Figure S3A: Cell survival of β-CTF/C6 cells vs. cumate doses, Figure S3B: Cell survival of β-CTF/C6 cells vs. γ-secretase inhibitor doses, Figure S4: Cell survival rate of rat primary cortical neurons vs. Aβ42 concentration, Figure S5: Effect of various signaling pathway inhibitors on vitamin K2-dependent protection.

## Abbreviations

A6730	Akt 1/2 inhibitor
AD	Alzheimer’s disease
Aβ	Amyloid-β
Akt	Protein kinase B
ApoE4	Apolipoprotein E
APP	Amyloid precursor protein
Axl	Axl receptor tyrosine kinase
Bad	Bcl-2-associated death promoter protein, tyros3, Axl and Mer (TAM) family
C99	C-terminal 99 residues of APP
β-CTF	C-terminal 99-residue fragment of APP
β-CTF/C6	C6 cell line-containing β-CTF gene
ERK1/2	Extracellular signal-regulated protein kinases 1 and 2
Gas6	Growth arrest-specific protein 6
HEK293	Human embryonic kidney 293
c-JNK	C-Jun N-terminal kinase
LY294002	PI3K inhibitor
MAPK	Mitogen-activated protein kinase
MK4	Menaquinone-4
NF-κB	Nuclear factor kappa-light-chain-enhancer of activated B cells
p38-SAPK	Stress-activated protein kinase p38
PD98059	Extracellular signal-regulated protein kinases 1 and 2 (ERK) inhibitor
PI3K	Phosphatidylinositol 3-kinase
R428	Axl inhibitor
ROS	Reactive oxygen species
SB202190	Stress-activated protein kinase p38 (p38-SAPK) inhibitor
SN50	NF-κB inhibitor
SP600125	JNK inhibitor
VKD	Vitamin K-dependent
