# Supplementary material for: Molecular Mechanism of Vitamin K2 Protection against Amyloid-β-Induced Cytotoxicity
Source: Biomolecules. 2021 Mar 13;11(3):423. doi: 10.3390/biom11030423 (PMC8000266; doi:10.3390/biom11030423)
Supplement: Supplementary file 1 [file biomolecules-11-00423-s001.pdf]

biomolecules

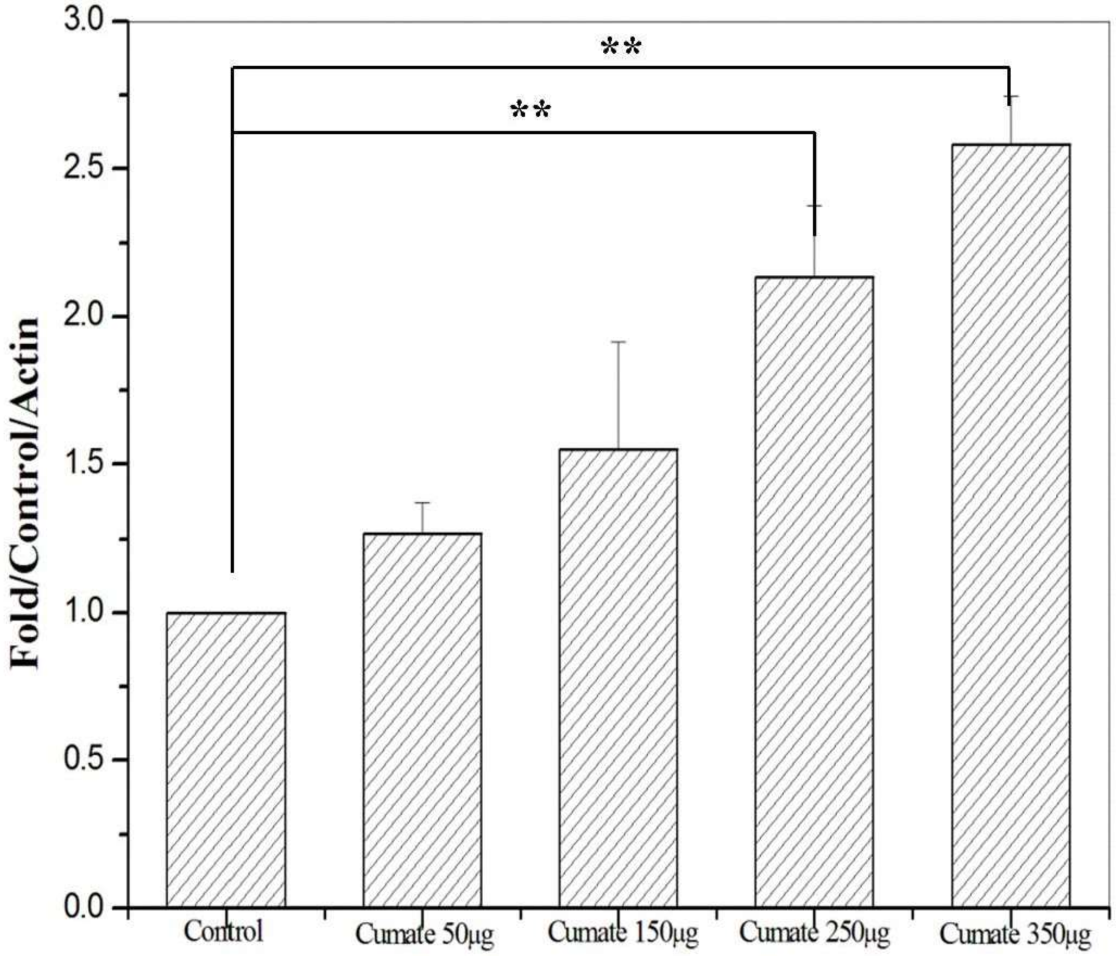

Figure S1. qPCR analysis of C99 expression vs. cumate doses. Data are mean ± SD. ** $p < 0.01$.

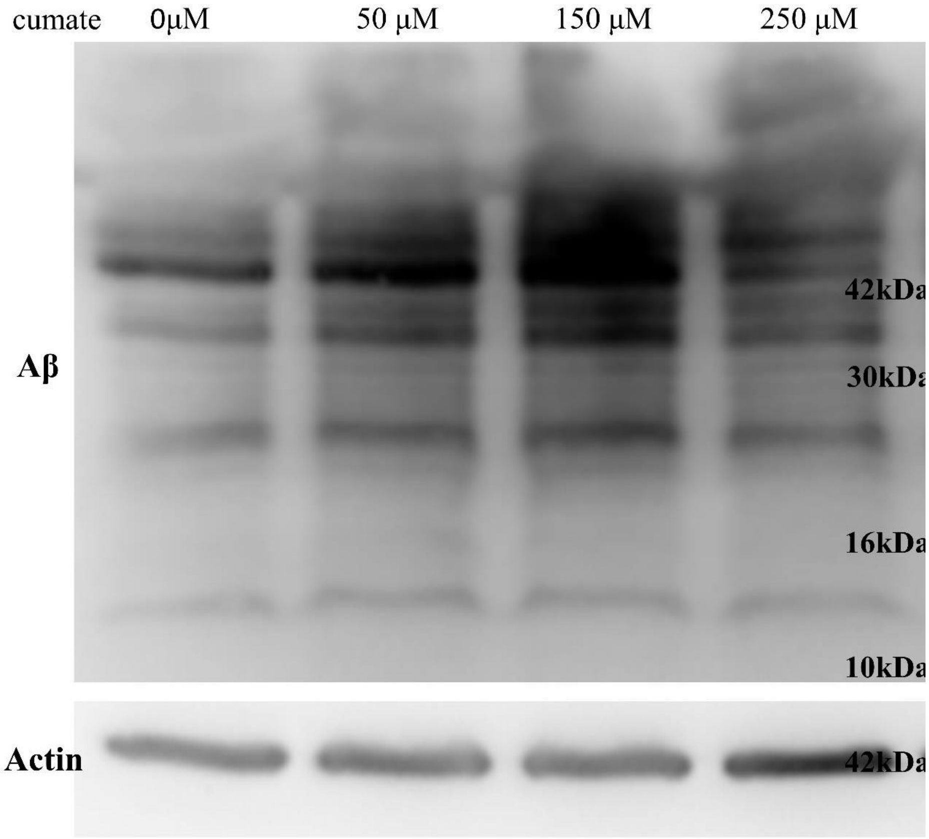

Figure S2. Western blot analyses of Aβ protein expression in βCTF/C6 cells treatedwith 0-250 μM of cumate.

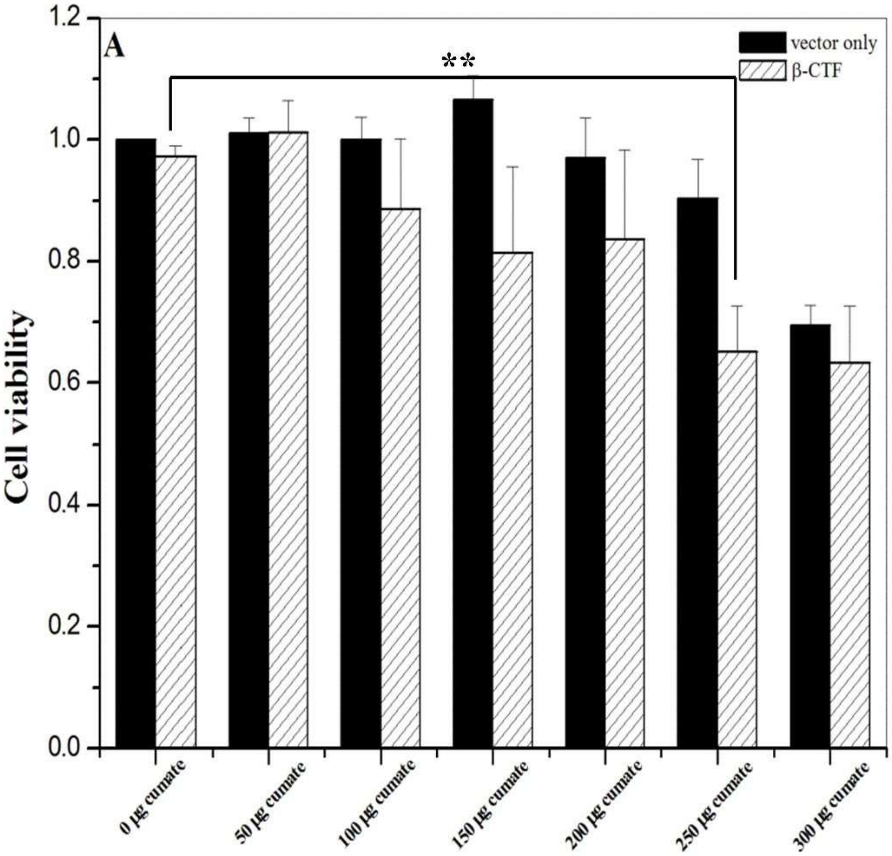

Figure S3. (**A**). Cell survival of β-CTF/C6 cells vs. cumate doses. The cell viability wasdecreased with an increase of cumate concentration and reached to ~60% survival rateat cumate concentration ≥ 250 μg. Data are mean ± SD. $^{**}p < 0.01$.

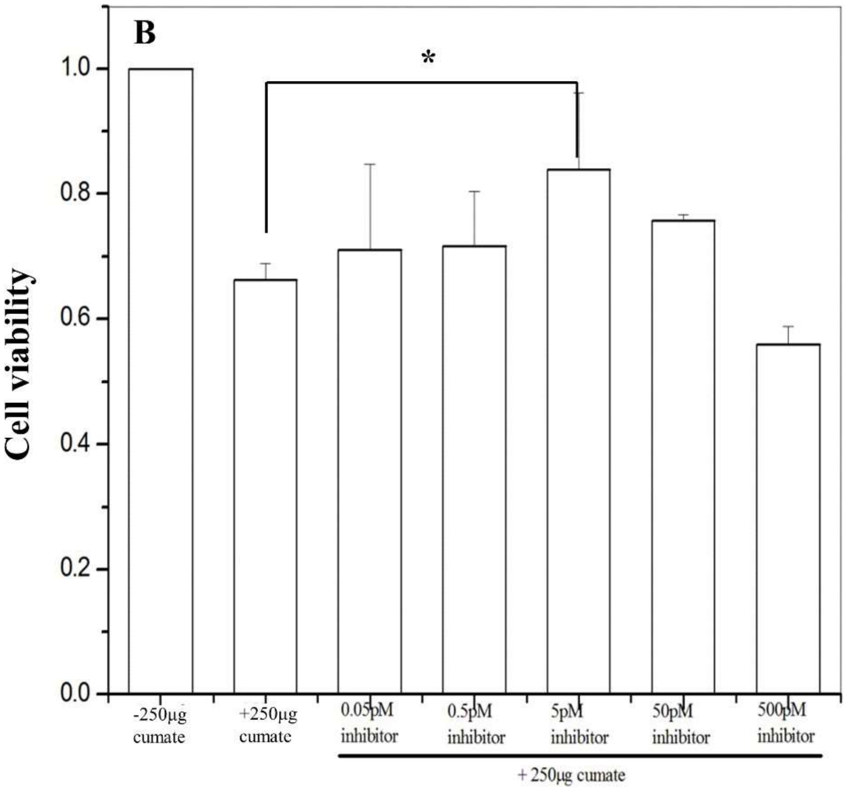

Figure S3. (**B**). Cell survival of β-CTF/C6 cells vs. γ-secretase inhibitor doses. The cell survival rate at 5 pM of γ-secretase inhibitor showed the most effective on rescue of cells against Aβ cytotoxicity, while the cell viability was reduced. The reason might be due that the concentration of γ-secretase inhibitor is overdosed. Data are mean ± SD.*$p < 0.05$.

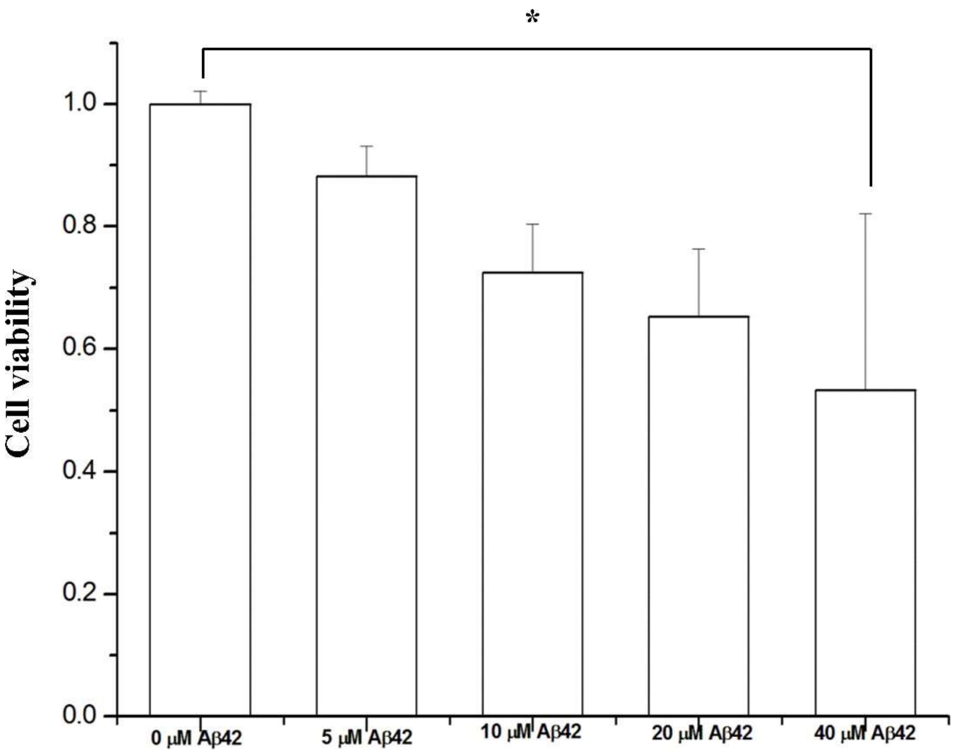

Figure S4. Cell survival rate of rat primary cortical neurons vs. Aβ42 concentration. The cell viability decreased with an increase of Aβ42 concentration. The cell survivalrate was 60% with treatment of 40 μM Aβ42, and thus concentration was then used totreat primary neurons in inhibitory assay. Data are mean ± SD. *$p < 0.05$.

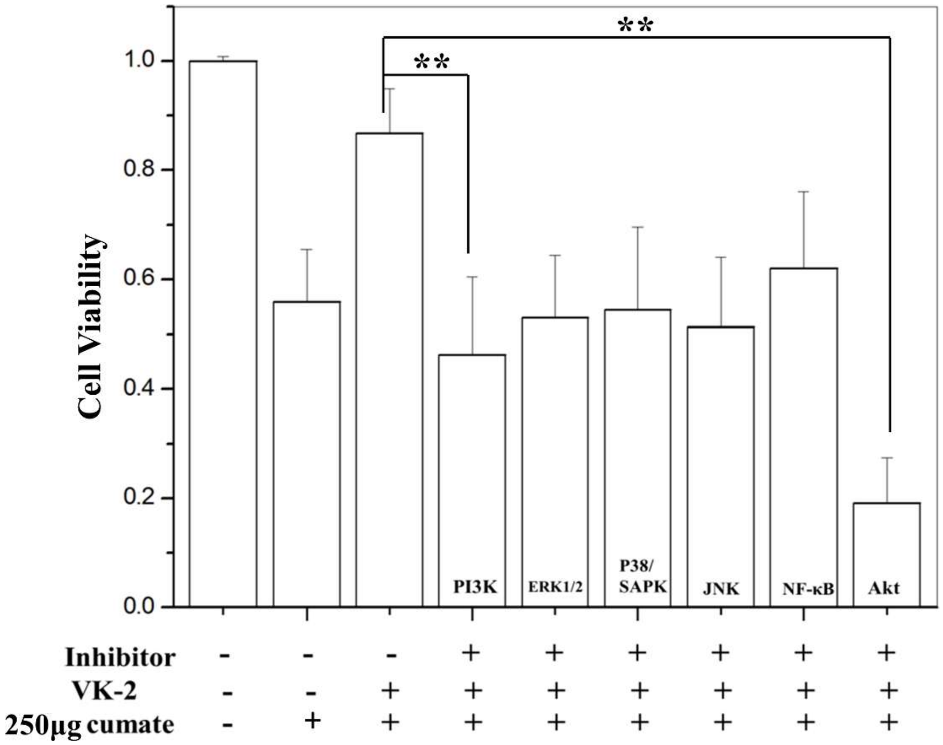

Figure S5. Effect of various signaling pathway inhibitors on vitamin K2-dependent protection. β–CTF/C6 cells were incubated with 10 μM inhibitors, 250 μg cumate and10 μM vitamin K2.The cell survival rate was determined by MTT assay. Data are mean ± SD. $^*p < 0.05$, and $^{**}p < 0.01$.
